# Necrotizing pneumonia in children: Chest computed tomography vs. lung ultrasound

**DOI:** 10.3389/fped.2022.898402

**Published:** 2022-08-26

**Authors:** Johann Carrard, Sebastien Bacher, Isabelle Rochat-Guignard, Jean-François Knebel, Leonor Alamo, Jean-Yves Meuwly, Estelle Tenisch

**Affiliations:** ^1^Department of Radiology, Riviera-Chablais Hospital, Rennaz, and University of Lausanne, Lausanne, Switzerland; ^2^Department of Radiology and Interventional Radiology, Lausanne University Hospital and University of Lausanne, Lausanne, Switzerland; ^3^Department Woman-Mother-Child, Unit of Pediatric Pulmonology, Lausanne University Hospital and University of Lausanne, Lausanne, Switzerland

**Keywords:** lung ultrasonography (LUS), pediatric pulmonology, chest ultrasound, necrotizing pneumonia, chest computed tomography (CT), children

## Abstract

**Background:**

The utilization of contrast-enhanced computed tomography (CT) of the chest for the diagnosis of necrotizing pneumonia (NP), a complication of community-acquired pneumonia, is controversial because of the inherent ionizing radiation involved. Over the past few years, the growing availability of bedside Lung Ultrasound (LUS) devices has led to increased use of this nonionizing imaging method for diagnosing thoracic pathology, including pneumonia.

**Objective:**

The objectives of this study were as follows: first, to compare the performance of LUS vs. CT in the identification of certain radiological signs of NP, and second, to determine whether LUS could replace CT in the diagnosis of NP.

**Materials and methods:**

We compared retrospectively the CT and LUS images of 41 patients between 2005 and 2018 in whom at least one contrast-injected chest CT scan and one LUS had been undertaken fewer than 7 days apart.

**Results:**

Pleural effusions were demonstrated almost systematically (100% on CT vs. 95.8% on LUS). Visualization of septations in pleural effusions was clearly superior on LUS (20.4% on CT vs 62.5% on LUS). Concerning the detection of necrosis, we observed a strong correlation between LUS and the gold-standard CT (95.8% on LUS vs. 93.7% on CT). Parenchymal cavities were more easily detected on CT than on LUS (79.1 vs. 35.4%).

**Conclusion:**

LUS has shown to be as effective as CT in the diagnosis of NP. The use of CT in patients with NP could be limited to the detection of complications such as bronchopleural fistulae in unfavorably evolving diseases.

## Introduction

Necrotizing pneumonia (NP) is a relatively frequent complication of community-acquired pneumonia, with up to 7% of pneumonia evolving to necrotizing pneumonia (1). The majority of patients have no significant prior medical history. The clinical picture of NP is initially similar to the presentation of community-acquired pneumonia. However, patients with NP as opposed to simple pneumonia are often seen to rapidly deteriorate, often associated with a prolonged fever (2, 3).

Radiological imaging is necessary for the diagnosis of NP. The typical CT signs include the loss of normal pulmonary parenchymal architecture and the presence of areas of decreased parenchymal enhancement, representing liquefaction, that are progressively replaced by multiple small air or fluid-filled cavities (2, 4). The pathophysiology of NP is thought to be massive pulmonary gangrene, tissue liquefaction, and necrosis, often associated with empyema (2, 5). At first, the lesions are not obvious on conventional X-rays but appear progressively over 5–9 days (4). Progressive pulmonary necrosis then evolves to form cavities or pneumatoceles, occasionally causing bronchopleural fistulas (6) the most frequent complication of the disease. Over the past few years, the wide availability of bedside Lung Ultrasound (LUS) devices in emergency centers has led to an increasing use of this non-ionizing form of imaging for the diagnosis of thoracic pathology, including that of pediatric pneumonia (7, 8).

To date, most published studies that have assessed the performance of LUS in diagnosing community-acquired pneumonia in children have compared LUS with conventional X-ray (9). Concerning NP, a study undertaken by Lai et al. (10) compared LUS with CT, demonstrating a strong correlation between the two diagnostic methods. However, the study compared lung perfusion on Doppler-LUS with the extension of necrotic parenchyma on CT. Moreover, LUS in this publication were performed by a very experienced operator. As X-ray remains the primary modality of imaging in suspected pediatric lung infection, we wanted to compare second-line modalities, specifically LUS (performed as a point-of-care ultrasound) and CT.

The objectives of this study were as follows: first, to compare the performance of LUS vs. CT in the identification of radiological signs of NP, including the detection of necrotic lung parenchyma, and second, to determine whether LUS could replace CT in the diagnosis of NP, even if performed by a junior radiologist or in a point-of-care setting.

## Materials and methods

Authorization to conduct this study was obtained from the Ethics Commission for Research on Humans (CER-VD).

A retrospective search covering 1 January 2005 to 28 February 2018, utilizing the key words “necrotizing pneumonia” and “chest ultrasound,” was undertaken using the hospital's radiology database. The study's inclusion criteria were as follows: patients <18 years old with a clinical and radiological diagnosis of NP, in whom at least one contrast-injected chest CT scan and one LUS had been done fewer than 7 days apart. Excluded from the study were patients whose LUS was not of sufficient quality in order to analyze the pulmonary parenchyma (even if demonstrating a pleural effusion: two cases with missing US images) and those in which LUS and CT were performed >7 days apart. Cases of non-pneumonia-related cavities, such as tuberculosis, septic pulmonary emboli, congenital malformations of the respiratory tract, and acute chest syndrome, were also excluded (diagnosis at hospital discharge).

When more than one LUS was available, the LUS and CT performed within the shortest period were selected. The mean time between exams was 1.7 days (range: 0–6 days; median: 2 days). Each examination was read by two radiologists, one senior (>10 years of experience, ET) and one junior (<1 year of experience, JC). To avoid selection bias, the chest CT and LUS of 20 other children of matching age (mean age: 81.6 months; median age: 56 months) having diagnosed pathologies other than NP, performed <3 days apart, were also examined.

### Chest computed tomography

The CT exams were performed on a General Electric (GE) 64-slice VCT system (General Electric Medical System, Milwaukee, WI, USA). Acquisitions were obtained after the intravenous injection of 1.5–2 ml/kg of a non-ionic contrast medium (Accupaque 300, GE Healthcare, Giles, UK) followed by flushing with 10–20 ml of 0.9% saline solution. The administration speed varied from 0.5 to 1 ml/second depending on the age of the patient and the venous access. Acquisition time varied between 20 and 25 s after injection. The helical acquisition was performed with free breathing for uncooperative children and with breath holding when possible. Table 1 summarizes the protocols used and the radiation doses administered to the patients.

**Table 1:** Protocols and doses of chest CT.

	**Age (years)/weight (kg)**		
**Thorax**	**1–6/10–25**	**6–12/25–40**	**>12/>40**
Scout view AP+ lateral 80 kV, 10 mA	+	+	+
Gantry rotation time (s)	0.5	0.5	0.5
Pitch	1.375	1.375	1.375
Slice thickness (mm) nom/rec	0.625/2.5	0.625/5 (2.5)	0.625/5 (2.5)
kV/mA	100/160	120/120–180	120/180
CTDIw (mGy)	2.59	3.92	4.06
DLP (mGycm)	58.87	156.4	134.3
Matrix size	512×512	512×512	512×512
FOV (mm)	240	240	240

We retained the following criteria for the analysis of the CT:

1. Pleura: the presence of pleural effusion, whether septated or not, pneumothorax, hydropneumothorax or bronchopleural fistula, and the chest drain *in situ*.
2. Parenchyma: the absence of contrast enhancement within the parenchymal condensations corresponding to necrosis, the presence of cavities, and atelectasis.

### Lung ultrasound

LUS was carried out on an IU22 Philips Healthcare machine with a L-12-5 probe or a C9-2 probe for larger patients. Protocol: Scanning and realization of at least two anterior images, two posterior images, and one on the axillary line, on both sides. If pathological images were produced, more images would be produced.

The LUS assessment criteria were as follows:

1. Pleura: the presence of pleural effusion, whether septated or not, hydropneumothorax, and the chest drain *in situ*.
2. Parenchyma: the presence of a heterogeneous hypoechogenic consolidation containing more hypoechogenic confluent lesions corresponding to necrotic cavities.

### Statistical comparison of chest CT and LUS

The relationship between LUS and CT features of NP were compared using descriptive statistics.

The diagnosis of lung necrosis on LUS made by a senior radiologist and a junior radiologist (blinded to the CT results) was evaluated with a kappa Cohen test, including patients with and without NP. No false-positive LUS (for necrosis) was reported (even if 20 cases without necrosis were included in the study). Therefore, a sensitivity/specificity comparison between CT and LUS regarding the diagnosis of parenchymal necrosis could not be done.

All discordant findings between CT and LUS (i.e., false-negative on LUS) were analyzed and reviewed, specifically those reporting the presence of parenchymal necrotic cavities, with or without air-fluid levels. To determine the cause of each discrepancy, variables such as (1) the location of the cavity (deep parenchymal or superficial); (2) the size of any necrotic zones; (3) the number of necrotic cavities, and (4) the quality of the acoustic window were tested in accordance with incorrect/correct detection by LUS. The incorrect/correct detection was defined using CT as the gold-standard. This analysis was performed using chi-squared or Fisher's exact tests depending on the number of levels of each variable (two levels for Fisher's exact test and >2 levels for chi-squared test). Results were reported as numbers of correct/incorrect detections within each level of the variable and the *p*-value of each test, where a threshold of alpha <0.05 was considered significant. Statistical analyses were performed using Anaconda version 5·3·1 (Anaconda, Austin, Texas, United States) for Python 3.5 (PSF, Delaware, United States) linked *via* module Rpy2 with R version 3·5·1 (R Foundation, Vienna, Austria).

## Results

Initially, 48 children fulfilled the inclusion criteria but seven patients were excluded as a result of *Mycobacterium tuberculosis* infection (*n* = 3), the presence of septic pulmonary emboli (*n* = 1), congenital malformations of the respiratory tract (*n* = 1), and acute chest syndrome (*n* = 2). Finally, 41 patients (16 males and 25 females) met the inclusion criteria. The mean age of the patients was 58.7 months (range 17–182 months; median 46 months). In total, 35 had one CT/LUS pair, five patients had two CT/LUS pairs, and one had three CT/LUS pairs. A total of 48 pairs of examinations performed in 41 patients with NP were available. *Streptococcus pneumoniae* was the most frequent causative pathogen (76%) for cases where blood culture or urinary antigen was positive.

Then, 20 CT/LUS pairs of matching age with a different diagnosis (such as tumor and uncomplicated pneumonia) were also randomly selected for the interobserver variability. They were analyzed mixed with PN cases to avoid the bias of having only patients with NP.

### Chest CT

**Figure 1:**
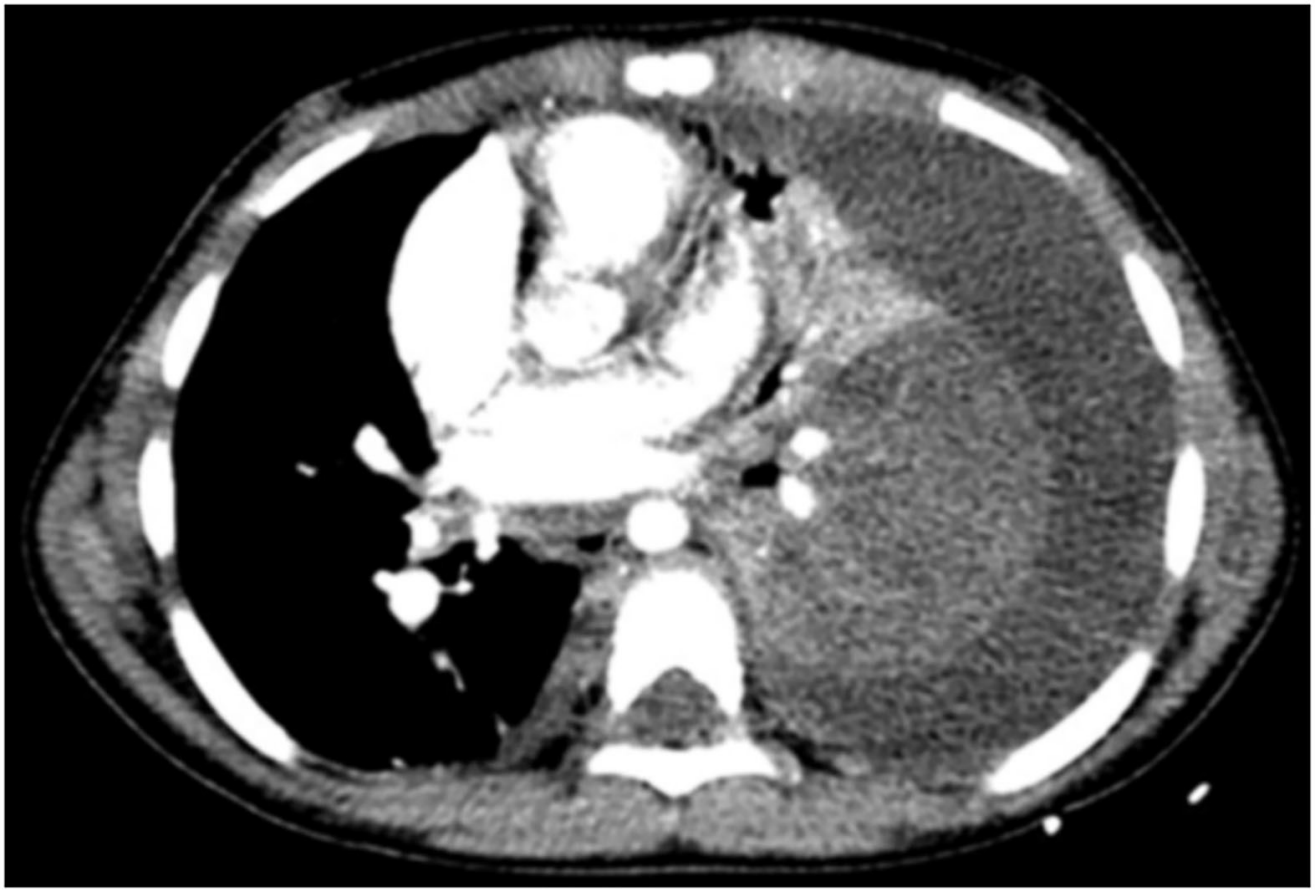
A 5-year-old patient with NP. Transverse slice of chest CT with injection in the mediastinum window shows heterogeneous enhancement of the parenchyma of the left lower lobe in keeping with NP before appearance of cavities. Also visible is a left pleural effusion causing partial atelectasis of the left lung.

1. Pleura: septated pleural effusions were present in 10 cases (20.8%) and non-septated effusions in 38 cases (79.2%); seven cases (14.6%) demonstrated pneumothorax and 10 (20.4%) hydropneumothorax. Bronchopleural fistula was present in seven cases (14.6%), taking into account the fact that 22.9% of the CTs were performed after chest drainage.

2. Lung parenchyma: forty-five cases (93.8%) presented unenhanced consolidation of the lung parenchyma after injection of a contrast agent. In 17 cases (35.4%), the necrosis was located in the left lower lobe (Figure 1); in 18 cases (37.5%), it involved the two lobes of the left lung; and in eight children (16.7%), the three lobes of the right lung. Cystic cavities were found in 38 cases (79.2%) and atelectasis of the necrosis lung in 42 cases (87.5%).

### Lung ultrasound

**Figure 2:**
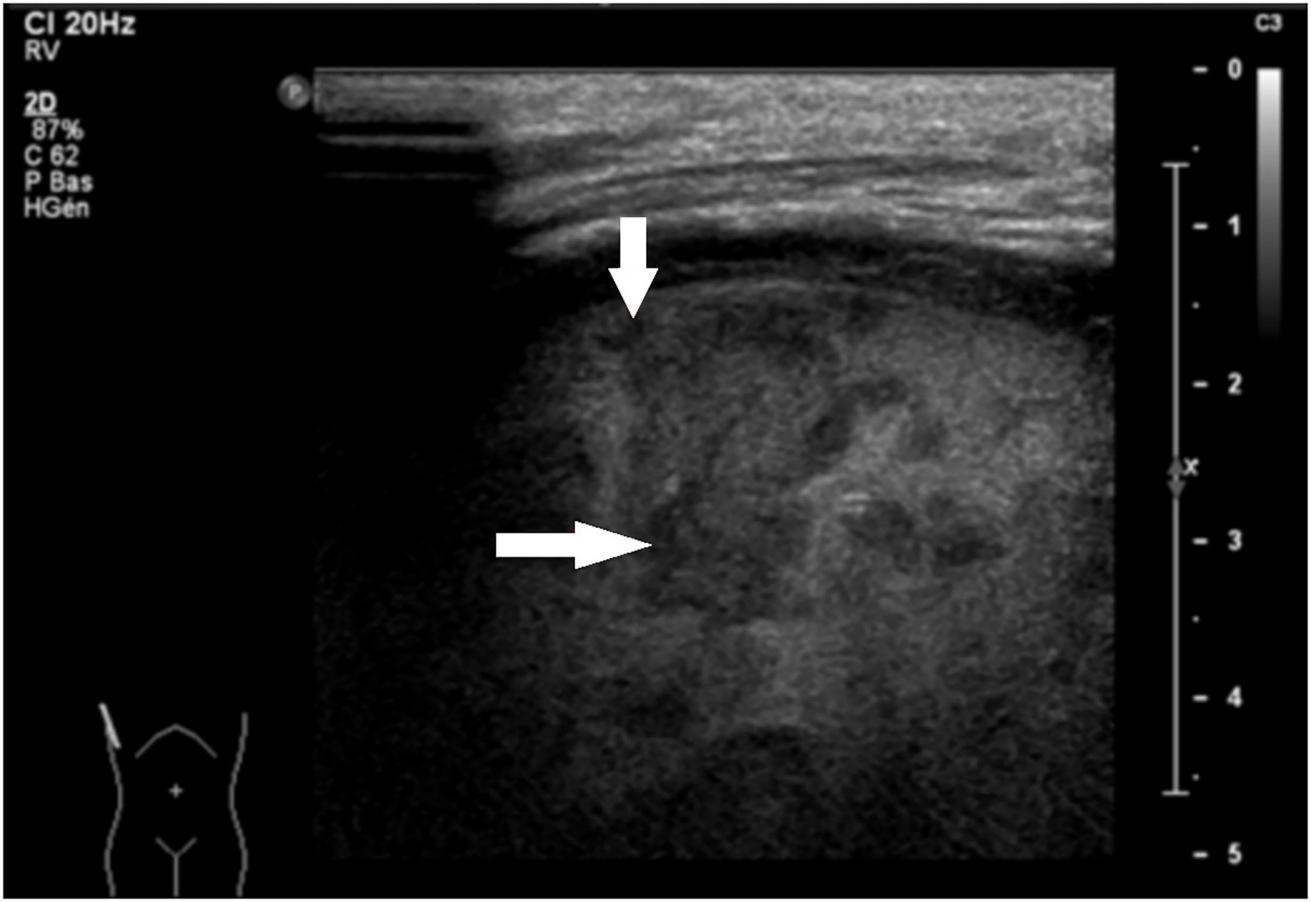
Sagittal LUS view of the right lung in an 8-year-old patient with fever and dyspnea. Heterogeneity of the right lung parenchyma corresponding to necrosis areas is clearly visible.

1. Pleura and pericardium: a total of 46 cases had visible pleural effusions (95.8%); 30 (62.5%) of these were septated and the remaining 16 were non-septated (27.5%). In addition, six cases (12.5%) presented a hydropneumothorax. A chest drain was visible in five cases (10.4%). Among these five cases, three had a (hydro-) pneumothorax identifiable on LUS Images.

2. Lung parenchyma: consolidation with necrotic areas and developing cavities in the lung was present in 46 cases (95.8%) (Figure 2), and atelectasis (in adjacent lobes) was depicted in 33 cases (68.8%).

### Comparison between computed tomography and ultrasound

Table 2 compares the results obtained on CT and LUS.

**Table 2:** Comparison between chest computed tomography and lung ultrasound detection of features in patients with necrotizing pneumonia (48 pairs of exams in 41 patients).

**Radiological Sign**	**Ultrasound (%)**	**CT (%)**
Heterogeneous consolidations	95.8	93.7
Atelectasis	68.7	87.5
Cystic cavities	35.4	79.1
Septated pleural effusions	62.5	20.4
Non-septated pleural effusions	33.3	79.1
Hydropneumothorax	12.5	20.4
Drains	11.1	22.9
Bronchopleural fistulae	0	14.6

**Figure 3:**
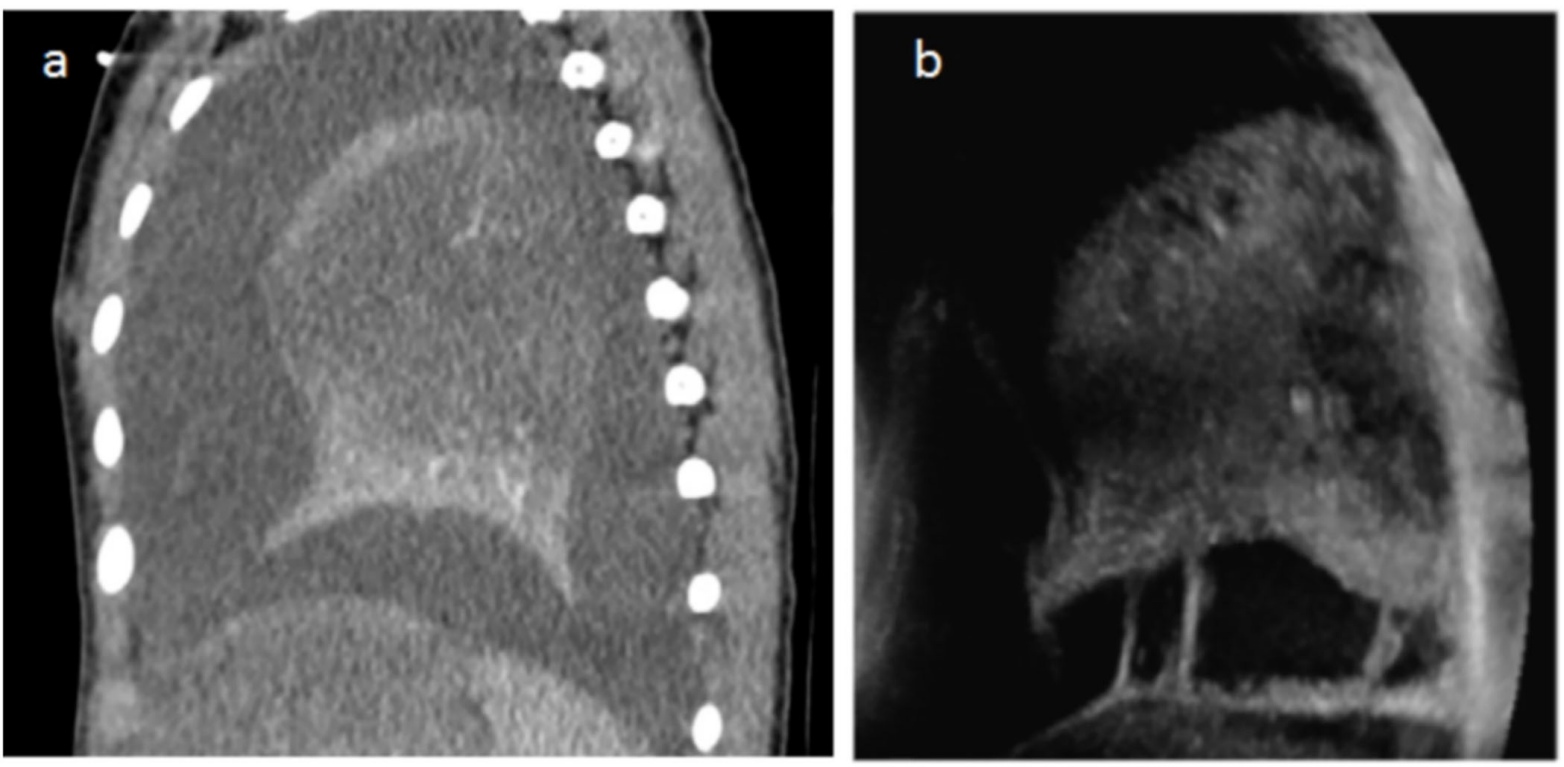
**(a)** Chest CT with contrast agent injection in a 2.5-year-old child with 39°C fever, cough, and breathing difficulties. Sagittal reconstructions show a heterogeneous lung with unenhanced parenchyma corresponding to necrosis and a large pleural effusion. The US performed the same day also shows heterogeneity and hypodensity of the parenchyma in the necrotic zones (same as on CT) and pleural effusion. The periphery of the lower lobe is spared in a similar way on LUS and CT. In addition, US demonstrates bands of fibrin within the effusion, not visible on CT **(b)**.

**Figure 4:**
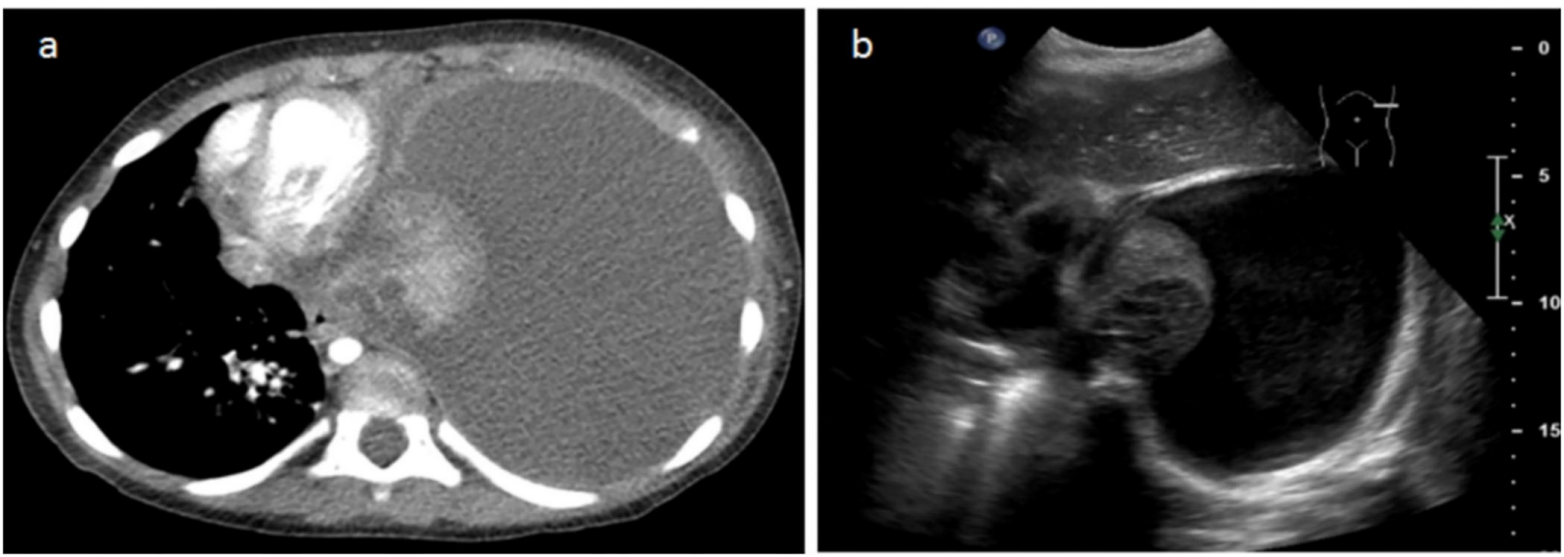
A 3-year-old female patient with severe dyspnea. **(a)** The transverse slice chest CT demonstrates massive left effusion and atelectasis of the entire left pulmonary parenchyma, with heterogeneous enhancement and round necrotic unenhanced lesions posteriorly. **(b)** LUS performed the same day demonstrates the same parenchymatous damage with coalescent cystic lesions corresponding to necrosis.

We found an excellent correlation between the two techniques in the evaluation of the pulmonary parenchymal disease—areas of consolidation and delimitation of necrosis (Figures 3, 4). As described in the statistics section, a comparison of the specificity and sensibility of the LUS with the gold standard (i.e., chest CT) for the detection of lung necrosis was not feasible because there were no false-positive LUS. LUS performed better than CT in identifying septations within the pleural effusions (Figure 3), whereas CT allowed a better visualization of (hydro-)aeric cavities and atelectasis.

### Interobserver variability

Analysis of patients with (41 patients = 48 CT-LUS pairs) and without NP (20 patients of matching age).

When interpreting the LUS images for the detection of lung necrosis, the senior radiologist had an almost perfect accuracy according to the CT results (kappa = 0.895) and the junior radiologist had a moderate accuracy (kappa = 0.49). The uniformity between the radiologists' reports was measured as moderate with a kappa = 0.568.

### Variables influencing the detection of lung necrosis on LUS

The number of necrotic zones and the acoustic window did not significantly influence the detection of pulmonary necrosis (*p* = 0.117 and 0.101, respectively). In contrast, the depth of the necrotic zones within the lung parenchyma and the size of the necrotic areas played a significant role in their detection by LUS (*p* = 0.082) for both variables.

## Discussion

Nearly 7% of all community-acquired pneumonias are necrotizing (1).

A review of the literature demonstrates an increase in the incidence of this pathology in children (1, 3), which may be partly explained by the increasing use of CT in the diagnosis of pediatric chest infections over the last decade.

The series that we report here assessed 41 patients <18 years of age with NP over a 13-year period with 48 CT/LUS combinations. Pleural effusions were found almost systematically (100% on CT vs. 95.8% on LUS). The systematic presence of large pleural effusions at the time of diagnosis lends a true advantage to the LUS technique, as the excellent acoustic window provided by the pleural fluid allows a thorough evaluation of the underlying lung parenchyma. This observation favors the more frequent usage of LUS in the early phase of the disease (9, 11), before a drain is inserted.

When comparing CT and LUS in the visualization of septations in pleural effusions, we are in agreement with Trinavarat et al. (12) who describe the clearly superior capacity of LUS (62.5% on LUS vs. 20.8% on CT), relevant in particular before deciding on whether to place a pleural drain and where to position it. However, in a few cases, the CT can be very helpful in deciding whether air-containing lesions correspond to pneumatocele or to a loculated hydro-pneumothorax. In our series, we found bronchopleural fistulae in 14.6% of cases, visible only on CT, a finding in accordance with the literature (2) and confirming the usefulness of CT in this type of situation. Bronchopleural fistulas were present in the areas of low-attenuation lung consolidation that appeared to communicate directly with an empyema or with a disruption of the visceral pleura; associated air in the pleural space was not always seen. This rare complication seems to have increased over the last decade, but it is probable that it follows the increased incidence of NP (13, 14). Another explanation could be that the insertion of a chest tube without ultrasound or CT guidance (as sometimes performed in our institution) may lead to lesions of the already very fragile necrosed lung, causing secondary bronchopleural fistulas. In our series, all cases of bronchopleural fistulas were managed conservatively, without surgery.

We were surprised to find that three cases in this series did not show parenchymal necrosis on CT, while necrosis itself defines NP. This is explained by the fact that, in these patients, CT was performed very early after the onset of the symptoms, but it was the evolution of the disease that confirmed the diagnosis later by chest X-ray or a second CT (with parenchymal cavities). In one patient, the LUS was performed on the same day as the CT already showed parenchymal necrosis, barely visible on CT.

Necrosis of lung parenchyma is usually rapidly visible on CT as a multifocal lack of enhancement of the affected areas after contrast injection (4). Concerning the detection of necrosis, we observed a strong correlation between LUS and CT (95.8% on LUS vs. 93.7% on CT). LUS shows a hepatized hypoechogenic parenchyma (relative to the adjacent hyperechogenic parenchyma with “simple” pneumonia or atelectasis) that becomes heterogeneous with small hypoechoic regions, converging to form geographic zones of necrosis corresponding to the non-enhancing zones on CT (1, 10). These regions may progress to anechoic cysts. If these areas of necrosis end up communicating with a ventilated bronchus, they may present an air-fluid level or they may evolve into pneumatoceles (2, 4, 15).

It should be noted that, in this retrospective study, different operators—mostly radiology residents at the beginning of their training—performed the LUS exams in an emergency setting and that, in most cases, the examinations were performed to search for pleural effusion and not specifically for pulmonary necrosis. Despite these limitations, the necrotic areas were clearly identifiable on the available images. It suggests that a point-of-care LUS could be useful for the follow-up of pulmonary necrosis, even in exams performed by junior radiologists (9).

LUS cannot quantify necrotic damage, particularly when regarding images retrospectively. However, the amount of necrosis does not seem to play a major role in the patients' evolution (16). Moreover, quantification of necrosis is sometimes difficult on CT due to frequent atelectasis.

Parenchymal cavities were more easily detected on CT than on LUS (79.1 vs. 35.4%). The false-negative results on LUS were explained by the location of the lesions. We observed that cavities situated relatively deep within the parenchyma were partially hidden by the more superficial and still normally aerated lung parenchyma. The atelectasis caused by pleural effusion was probably also limiting the visualization of necrosis in some situations.

An extensive meta-analysis claims that LUS has excellent specificity and sensitivity in the diagnosis of pediatric pneumonia (17). However, to our knowledge, few studies have attempted to assess the utility of LUS in the diagnosis of NP or compared LUS directly to CT (10, 18). This series brought together a large number of patients who underwent both LUS and CT and performed close together in time, allowing the comparison of the two methods. In view of our results, we consider that LUS should be highlighted in the diagnosis or in the follow-up of NP. Moreover, our study shows that even a junior operator can image lung parenchyma and detect parenchymal necrosis with a rapidly available imaging modality (even before a peripheral IV line is inserted), which should be taken into account in the era of point-of-care ultrasound. This is possible as long as the operator performing the ultrasound follows a structured protocol (11). The operator should keep in mind that necrosis predominates in the left lung and should search actively in this region, particularly in the case of pleural effusion with an infectious context. Moreover, we propose to begin with a medium or low-frequency probe in order to identify all the potential necrosis and then to analyze it with a high-frequency probe, depicting more precisely the cystic transformation of the parenchyma. In the case of doubt, a senior radiologist may interpret the findings retrospectively, as undertaken in this study.

LUS imaging contributes greatly in terms of radioprotection, in accordance with the widespread application of the ALARA principle and following the dictum “Image gently” (11). In addition, the intravenous injection of a contrast agent required to identify necrotic lung areas on CT adds the risk of allergic reaction and is limited in children with renal insufficiency (19).

This study has several limitations, the retrospective design being a limitation in itself. Moreover, the study compared CT with B-mode LUS exclusively, whereas the Doppler study of the parenchyma or the injection of an echographic contrast agent seemed to provide a better estimation of the extension of necrosis (10). However, Doppler-US and contrast-enhanced LUS are not performed in a point-of-care setting. Moreover, young patients with NP are often very tachypneic and restless, making Doppler imaging limiting and often inaccurate, even for an experienced operator. We hypothesize that a prospective study with systematic and complete LUS of both lungs would most likely obtain results closer to CT in terms of identifying parenchymal lung necrosis.

## Conclusion

LUS is an interesting tool for the diagnosis of NP. Its use should be encouraged, especially as the use of point-of-care LUS in the pediatric emergency departments is increasing. In the future, the use of CT in children with NP could be limited to the detection of complications such as bronchopleural fistulae in unfavorably evolving diseases.

## Data availability statement

The original contributions presented in the study are included in the article/supplementary material, further inquiries can be directed to the corresponding author/s.

## Ethics statement

The studies involving human participants were reviewed and approved by Commission cantonale d'éthique de la recherche sur l'être humain (CER-VD).

## Author contributions

JC and ET conceptualized and designed the study, collected the data, analyzed, drafted the initial manuscript, and reviewed the manuscript. SB acquired and analyzed the data. JC, ET, and SB revised the manuscript. LA contributed to the conception of the study, drafted the article, and revised it critically. IR-G analyzed and interpreted the data, and revised it critically from the clinical point of view. J-YM revised the manuscript from the imaging point of view. J-FK collected the data, designed the statistics, and critically reviewed the manuscript. All authors contributed to the article and approved the submitted version.

## Funding

Open access funding provided by University of Lausanne.

## Conflict of interest

The authors declare that the research was conducted in the absence of any commercial or financial relationships that could be construed as a potential conflict of interest.

## Publisher's note

All claims expressed in this article are solely those of the authors and do not necessarily represent those of their affiliated organizations, or those of the publisher, the editors and the reviewers. Any product that may be evaluated in this article, or claim that may be made by its manufacturer, is not guaranteed or endorsed by the publisher.
