# Erratum: Necrotizing pneumonia in children: Chest computed tomography vs. lung ultrasound

**DOI:** 10.3389/fped.2023.1178223

**Published:** 2023-03-23

**Authors:** Frontiers Production Office

**Affiliations:** Frontiers Media SA, Lausanne, Switzerland

**Keywords:** lung ultrasonography (LUS), pediatric pulmonology, chest ultrasound, necrotizing pneumonia, chest computed tomography (CT), children

An Erratum on Necrotizing pneumonia in children: Chest computed tomography vs. lung ultrasound By Carrard J, Bacher S, Rochat-Guignard I, Knebel J-F, Alamo L, Meuwly J-Y and Tenisch E. (2022) Front. Pediatr. 10:898402. doi: 10.3389/fped.2022.898402

An omission to the funding section of the original article was made in error. The following sentence has been added: “Open access funding was provided by the University of Lausanne”.

The original version of this article has been updated.

